# Supplementary material for: Mass Spectrometry-Based Peptide Profiling of Haemolymph from Pterostichus melas Exposed to Pendimethalin Herbicide
Source: Molecules. 2022 Jul 21;27(14):4645. doi: 10.3390/molecules27144645 (PMC9315633; doi:10.3390/molecules27144645)
Supplement: Supplementary file 1 [file molecules-27-04645-s001.zip › molecules-1813255-supplementary.pdf]

Table S1. Predicted neuropeptides by NeuroPIpred predictive tool (https://webs.iiitd.edu.in/raghava/neuropipred/).

ID	Seq	Score	Prediction	Hydrophobicity	Hydropathicity	Hydrophilicity
**FAR14_SARBU**	DPHHDFMRF	0.32528052	Yes	-0.29	-1.33	0.19
**NPF1_LEPDE**	ARGPQLRLRF	0.63979152	Yes	-0.40	-0.68	0.26
**ALLTR_ACRHI**	GFKNVALSTARGF	0.6016134	Yes	-0.06	0.23	-0.25
**ADFA_TENMO**	VVNTPGHAVSYHVY	-1.1651769	No	0.05	0.19	-0.86
**TXS6D_CUPSA**	INKYREWKNKKN	0.28297396	No	-0.63	-2.65	0.92
**TXS6D_CUPSA**	INKYREWKNKKN	0.28297396	Yes	-0.63	-2.65	0.92
**PPK_SCHGR**	DGAETPGAAASLWFGPRV	-0.2569061	Yes	-0.01	-0.02	-0.13
**ALL3_RHOPR**	QVSLKYPEGKMYSFGL	-0.43973767	Yes	-0.08	-0.31	-0.23
**BOL3_BOMPE**	IKIMDILAKLGKVLAHV	-1.7130195	No	0.08	1.18	-0.27
**BRK_VESMC**	TATTRRRGRPPGFSPFR	-0.78063843	No	-0.47	-1.39	0.51
**PH1_PERAM**	SDLTWTYQSPGDPTNSKN	0.39367822	Yes	-0.27	-1.55	0.10
**MK2B_PALPR**	VDKPDYRPRPWPRNMI	0.36148854	Yes	-0.43	-1.62	0.49
**LYC40_LYCSI**	IASHLAFEKLSKLGSKHTML	-0.85372578	No	-0.07	0.14	-0.11
**PAP2_SPOEX**	ENFAGGCTPGYQRTADGRCKPTF	-0.044915393	No	-0.24	-0.91	0.17
**PA11_VESVE**	GLLPKVKLVPEQISFILSTRENR	-0.8437491	No	-0.16	-0.06	0.10
**HN423_CYRHA**	DCAGYMRECKEKLCCSGYVCSSRWKWCVLPAP	1.7179663	No	-0.17	-0.17	-0.04
**MSPI2_MELSA**	EISCEPGTTFQDKCNTCRCGKDGKSAAGCTLKACPQ	0.79004272	Yes	-0.24	-0.62	0.36
**TXC1C_CUPSA**	GFGSLFKFLAKKVAKTVAKQAAKQGAKYIANKQTE	-1.1751568	No	-0.17	-0.34	0.16
**TALAA_DROME**	LDPTGTYRRPRDTQDSRQKRRQDCLDPTGQY	0.393764	Yes	-0.57	-2.07	0.85
**BX4_LOXGA**	ADSRKPDDRYDMSGNDALGDVKLATYEDNPWETFK	0.11494543	Yes	-0.33	-1.37	0.68
**CEC_CALVI**	GWLKKIGKKIGRVGQHTRDATIQGLAVAQQAANVAATAR	-1.9190137	No	-0.17	-0.26	0.06
**DIUH1_TENMO**	SPTISITAPIDVLRKTWEQERARKQMVKNREFLNSLN	1.000674	Yes	-0.30	-0.76	0.32
**LYC1_LYCSI**	GKLQAFLAKMKEIAAQTL	-0.91165539	No	-0.06	0.25	-0.06

ID	Seq	P			
**seq1**	DPHHDFMRF	**Yes**	FAR14_SARBU	FMRFamide-14	
**seq2**	ARGPQLRLRF	**Yes**	NPF1_LEPDE	Neuropeptide NPF-1	
**seq3**	GFKNVALSTARGF	**Yes**	ALLTR_ACRHI	ATRP; Allatotropin-related peptide	
**seq4**	VVNTPGHAVSYHVY	**No**	ADFA_TENMO	Antidiuretic factor A	
**seq5**	INKYREWKNKKN	**No**	TXS6D_CUPSA	Short cationic peptide-6d	
**Seq6**	DGAETPGAAASLWFGPRV-amide	**Yes**	PPK_SCHGR	Pyrokinin, Capa-Pk, Scg-PVK-3	+
**Seq7**	QVSLKYPEGKMYSFGL	**Yes**	ALL3_RHOPR	Allatostatin-3, Rhopr-AST-3	
**Seq8**	IKIMDILAKLGKVLAHV	**No**	BOL3_BOMPE	Bombolitin-3	+
**Seq9**	TATTRRRGRPPGFSPFR	**No**	BRK_VESMC	Vespulakinin-1	+
**Seq10**	SDLTWTYQSPGDPTNSKN	**Yes**	PH1_PERAM	Peptide hormone 1	
**Seq11**	VDKPDYRPRPWPRNMI	**Yes**	MK2B_PALPR	Metalnikowin-2B	+
**seq12**	IASHLAFEKLSKLGSKHTML	**No**	LYC40_LYCSI	M-lycotoxin-Ls4a	
**seq13**	ENFAGGCTPGYQRTADGRCKPTF	**No**	PAP2_SPOEX	Paralytic peptide 2	+
**seq14**	GLLPKVKLVPEQISFILSTRENR	**No**	PA11_VESVE	Phospholipase A1 verutoxin-1 (Fragment)	+
**seq15**	DCAGYMRECKEKLCCSGYVCSSRWKWCVLPAP	**No**	HN423_CYRHA	U3-theraphotoxin-Hhn1r	
**seq16**	EISCEPGTTFQDKCNTCRCGKDGKSAAGCTLKACPQ	**Yes**	MSPI2_MELSA	Serine protease inhibitor 2	
**seq17**	GFGSLFKFLAKKVAKTVAKQAAKQGAKYIANKQTE	**No**	TXC1C_CUPSA	Cupiennin-1c	
**Seq18**	LDPTGTYRRPRDTQDSRQKRRQDCLDPTGQY	**Yes**	TALAA_DROME	Peptide tarsal-less AA	
**Seq19**	ADSRKPDDRYDMSGNDALGDVKLATYEDNPWETFK	**Yes**	BX4_LOXGA	Dermonecrotic toxin LgSicTox-beta-LOXN4	+
**Seq20**	GWLKKIGKKIGRVGQHTRDATIQGLAVAQQAANVAATAR	**No**	CEC_CALVI	Cecropin	
**seq21**	SPTISITAPIDVLRKTWEQERARKQMVKNREFLNSLN	**Yes**	DIUH1_TENMO	Diuretic hormone 1	
